# Characterization of *Lactobacillus salivarius* strains B37 and B60 capable of inhibiting IL-8 production in *Helicobacter pylori*-stimulated gastric epithelial cells

**DOI:** 10.1186/s12866-016-0861-x

**Published:** 2016-10-18

**Authors:** Wimonrat Panpetch, Jennifer K. Spinler, James Versalovic, Somying Tumwasorn

**Affiliations:** 1 Interdisciplinary Program of Medical Microbiology, Graduate School, Chulalongkorn University, Bangkok, Thailand; 2 Texas Children’s Microbiome Center, Department of Pathology, Texas Children’s Hospital, Houston, TX USA; 3 Department of Pathology & Immunology, Baylor College of Medicine, Houston, TX USA; 4 Department of Microbiology, Faculty of Medicine, Chulalongkorn University, Bangkok, 10330 Thailand

**Keywords:** *Lactobacillus salivarius*, *Helicobacter pylori*, Interleukin-8, Anti-inflammatory, Probiotic

## Abstract

**Background:**

Interleukin (IL)-8 is the key agent for initiating an inflammatory response to infection with *Helicobacter pylori*. Some strains of *Lactobacillus* spp. are known to colonize the stomach and suppress inflammation caused by *H. pylori*. In this study, we characterized two gastric-derived lactobacilli, *Lactobacillus salivarius* (LS) strains B37 and B60, capable of inhibiting *H. pylori*-induced IL-8 production by gastric epithelial cells.

**Results:**

Conditioned media from LS-B37 and LS-B60 suppressed *H. pylori*-induced IL-8 production and mRNA expression from AGS cells without inhibiting *H. pylori* growth. These conditioned media suppressed the activation of NF-κB but did not suppress c-Jun activation. IL-8 inhibitory substances in conditioned media of LS-B37 and LS-B60 are heat-stable and larger than 100 kDa in size. The inhibitory activity of LS-B37 was abolished when the conditioned medium was treated with α-amylase but still remained when treated with either proteinase K, trypsin, lipase or lysozyme. The activity of LS-B60 was abolished when the conditioned medium was treated with either amylase or proteinase K but still remained when treated with lysozyme. Treatment with lipase and trypsin also significantly affected the inhibitory activity of LS-B60 although the conditioned medium retained IL-8 suppression statistically different from media control.

**Conclusions:**

These results suggest that *L. salivarius* strains B37 and B60 produce different immunomodulatory factors capable of suppressing *H. pylori-*induced IL-8 production from gastric epithelial cells. Our results suggest that the large, heat-stable immunomodulatory substance(s) present in the LCM of LS-B37 is a polysaccharide, while the one(s) of LS-B60 is either complex consisting of components of polysaccharide, lipid and protein or includes multiple components such as glycoprotein and lipoprotein.

**Electronic supplementary material:**

The online version of this article (doi:10.1186/s12866-016-0861-x) contains supplementary material, which is available to authorized users.

## Background

*Helicobacter pylori* is a well-known gastric pathogen which causes gastroduodenal inflammation, peptic ulceration and gastric cancer [1, 2]. *H. pylori* infection induces the production of pro-inflammatory cytokines and chemokines such as interleukin (IL)-1β, IL-6, IL-8, IL-23 and tumor necrosis factor (TNF)-α [3–5] resulting in gastric inflammation characterized by the infiltration of plasma cells, lymphocytes, neutrophils, and monocytes within gastric mucosa [5, 6]. IL-8 secreted by gastric epithelial cells is a potent neutrophil-activating and chemotactic agent [7, 8] which plays a major role in triggering the mucosal inflammation caused by *H. pylori* [9–13]. Increased levels of IL-8 in gastric juice and biopsy samples have been reported in patients with *H. pylori* infection [10, 11]. In addition, the levels of IL-8 mRNA in the gastric mucosa of *H. pylori*-infected patients correlate significantly with the severity of gastritis [14, 15] and the risk of gastric cancer [16].

Symptomatic subjects diagnosed with *H. pylori* infection generally receive eradication therapy. However, bacterial resistance to antibiotics and side effects which contribute to poor patient compliance result in suboptimal eradication rates [17, 18]. Probiotics have been shown to confer beneficial effects and are recommended as an adjunct in the treatment of *H. pylori* [18, 19]. Suppression of pro-inflammatory cytokine secretion by gastric epithelial cells is a mechanism of probiotic action which has been shown by numerous reports [20, 21] and considered as an approach to prevent gastric cancer [22]. *L. salivarius* UCC118 inhibited *H. pylori*-induced IL-8 production of AGS gastric epithelial cell by decreasing the function of the Cag secretion system [23]. *L. acidophilus* LA5® was shown to reduce IL-8 production induced by *H. pylori* in MKN45 gastric epithelial cells by inactivating the Smad7 and nuclear factor- kappa B (NF-κB) pathways [24]. Moreover, *L. gasseri* OLL2716 (LG21) was found to suppress *H. pylori*-induced IL-8 production in MKN45 gastric epithelial cells and decrease the level of IL-8 in the gastric mucosa of *H. pylori*-infected patients [25]. We previously reported eight isolates of *Lactobacillus* spp. which inhibited IL-8 secretion from *H. pylori*-infected AGS cells [26]. Among these lactobacilli, *L. salivarius* B101, *L. rhamnosus* B103 and *L. plantarum* XB7 suppressed IL-8 mRNA expression and the activation of NF-κB whereas *L. plantarum* XB7 also suppressed c-Jun activation. *L. salivarius* B37, *L. salivarius* B60 and the other three strains inhibited the secretion of IL-8 but did not interfere with IL-8 gene transcription after co-culture for 4 h with AGS cells.

In this study, we characterized the mechanism by which the previously identified *L. salivarius* B37 and B60 strains suppress IL-8 production from *H. pylori*-induced gastric epithelial cells. We hypothesized that *L. salivarius* B37 and B60 may suppress IL-8 gene expression at other time points or affect IL-8 production post-transcriptionally or post-translationally. The results of this present study demonstrated that *L. salivarius* B37 and *L. salivarius* B60 produce distinct active components that inhibit NF-κB activation and suppress downstream transcription of *H. pylori*-induced IL-8 mRNA.

## Results

### Gastric-derived *L. salivarius* strains B37 and B60 suppress IL-8 production in *H. pylori*-induced AGS cells

**Fig. 1:**
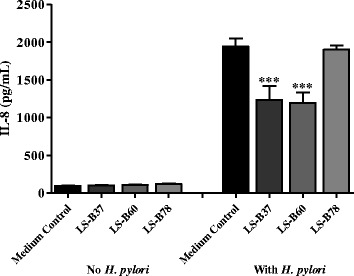
Specific strains of gastric-derived *L. salivarius* suppress IL-8 production by *H. pylori*-induced AGS cells. LCM from three strains of *L. salivarius* were tested for the ability to suppress IL-8 production from AGS cells stimulated by *H. pylori*. AGS cells were incubated with LCM in the presence or absence of *H. pylori* ATCC43504 for 24 h and IL-8 secretion was measured by ELISA. The results were from three independent experiments in triplicate and expressed as the mean ± SEM, ***p < 0.001 as compared to medium control

*L. salivarius* B37 (LS-B37) and *L. salivarius* B60 (LS-B60) are different strains as determined by random amplified polymorphic DNA (RAPD) and repetitive-sequence-based PCR (rep-PCR) fingerprinting (data not shown). *L. salivarius* B78 (LS-B78), which does not suppress IL-8, was included as a negative control. Immunomodulatory activity of substances from *Lactobacillus* conditioned media (LCM) was investigated by co-incubation with AGS cells either alone or in combination with viable *H. pylori* ATCC 43504. LCM of both LS-B37 and LS-B60 significantly (*p* < 0.001) suppressed IL-8 production in *H. pylori*-stimulated AGS cells, where LCM of LS-B78 did not suppress IL-8 production as expected (Fig. 1, Additional file 1). Trypan blue dye exclusion (>90 %, data not shown) indicated that IL-8 suppression by LS-B37 and LS-B60 was not due to cytotoxicity on AGS cells. LCM from LS-B37 and LS-B60 did not stimulate IL-8 production when co-incubated with AGS cells in the absence of *H. pylori* (Fig. 1, Additional file 1). In addition, 5 % v/v LCM in RPMI medium did not inhibit the growth of *H. pylori* (data not shown).

### Secreted factors from *L. salivarius* strains B37 and B60 diminished IL-8 gene expression

**Fig. 2:**
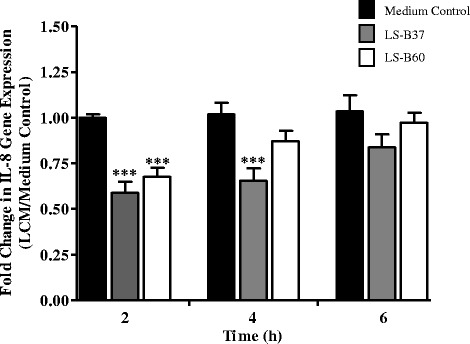
*L. salivarius*-produced factors suppress IL-8 transcription in AGS cells. IL-8 gene expression was determined in *H. pylori*-induced AGS cells after incubation with medium control or LCM from *L. salivarius*. Gene expression was examined using qRT-PCR with primers specific to IL-8 and GAPDH transcripts. Quantitative gene expression data were normalized to housekeeping gene, GAPDH. The experiments were performed three times independently, each in triplicate. Fold change of IL-8 gene expression was calculated in relative to medium control, and results represent the mean ± SEM, ***p < 0.001 as compared to medium control

Quantitative reverse transcription polymerase chain reaction (qRT-PCR) was used to determine the effect of LCM on IL-8 gene transcription. Since it was previously shown that LCM of LS-B37 and LS-B60 did not suppress IL-8 transcription after co-incubation with AGS cells for 4 h [26], the suppressive effect of these LCM was tested further at various time points. *H. pylori*-stimulated AGS cells were treated with LCM for 2, 4 and 6 h prior to total RNA isolation. IL-8 gene expression at 2 h, relative to *gapdh,* was significantly down-regulated approximately 0.4 and 0.3 fold with the treatment of LCM from LS-B37 (*p* < 0.0001) and LS-B60 (*p* < 0.0001), respectively. Unexpectedly, LCM from LS-B37 also down-regulated IL-8 gene expression approximately 0.3 fold (*p* < 0.0007) at 4 h. In addition, LCM from both strains did not affect IL-8 gene expression at 6 h (Fig. 2, Additional file 2).

### Secreted factors from *L. salivarius* strains B37 and B60 inhibit NF-κB activation

**Fig. 3:**
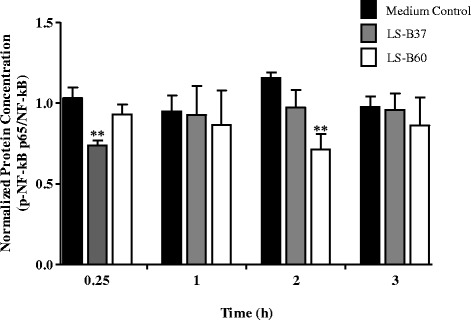
*L. salivarius* strains suppress *H. pylori*-activated NF-κB in AGS gastric epithelial cells. *H. pylori*-stimulated AGS cells were incubated with medium control or LCM of LS-B37 and LS-B60 for 0.25, 0.5, 1, 2, and 3 h. Concentration of activated NF-κB were measured by Western blot analysis using antibodies to p-NF-κB p65 and NF-κB p65. Relative protein concentration was quantified by densitometry and activated transcription factor (p-NF-κB p65 [Ser 536]) was normalized to non-activated counterpart (NF-κB p65). The results were from three independent experiments and expressed as the mean ± SEM, **p < 0.01 as compared to medium control

*H. pylori* induces various signaling pathways resulting in phosphorylation of transcription factors NF-κB and activator protein-1 (AP-1) and downstream transcription of IL-8 in gastric epithelial cells [27–29]. To determine the effect of immunomodulatory substances produced by LS-B37 and LS-B60 on the activation of NF-κB and AP-1, the concentrations of phosphorylated subunits p65 (p-NF-κB p65) and c-Jun (p-c-Jun) were assayed by Western blot. *H. pylori*-stimulated AGS cells were treated with LCM from either LS-B37 or LS-B60 for 0.25, 0.5, 1, 2 and 3 h. Treatment of LCM from LS-B37 decreased the concentration of p-NF-κB p65 at 15 min (28.52 %, *p* < 0.01, Fig. 3, Additional file 3) and no significant effects were found at other time points. Treatment with LS-B60 LCM resulted in a significant decrease in p-NF-κB p65 (38.29 %, *p* < 0.01, Fig. 3, Additional file 3) only at 2 h. In contrast, LCM from both strains did not suppress p-c-Jun at any studied time point (data not shown).

### The immunomodulatory substances in LCM of *L. salivarius* strains B37 and B60 are heat-stable and larger than 100 kDa

**Fig. 4:**
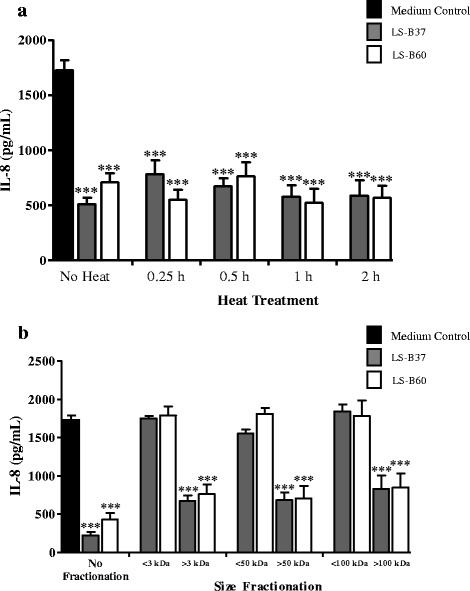
IL-8 suppression by heat-treated and fractionated LCM of *L. salivarius*. LCM of LS-B37 and LS-B60 were either heat treated (**a**) or fractionated by using Amicon centrifugal filter (**b**) and tested for the suppression of IL-8 production using a Quantikine Human IL-8 Immunoassay. The results were from three independent experiments in triplicate and expressed as the mean ± SEM, ***p < 0.001 as compared to medium control

Heat treatment and size fractionation of LCM were performed in order to characterize the physical nature of the IL-8 inhibitory substances. LCM from LS-B37 and LS-B60 were heated to 100 °C for 0.25, 0.5, 1 and 2 h and assayed for IL-8 suppression by an enzyme-linked immunosorbent assay (ELISA). Heat-treated LCM of LS-B37 and LS-B60 at all time retained inhibitory activity of *H. pylori*-induced IL-8 production in AGS cells when compared with MRS control (*p* < 0.001, Fig. 4a, Additional file 4).

Amicon® Centrifugal Filters (3 kDa, 50 kDa and 100 kDa) were used to size fractionate LS-B37 and LS-B60 LCM and each fraction was tested for inhibitory activity on *H. pylori*-induced IL-8 production. Fractions containing substances >100 kDa significantly suppressed IL-8 production as compared with MRS control (*p* < 0.001), whereas those containing substances <3 kDa, <50 kDa, and <100 kDa did not suppress *H. pylori*-induced IL-8 production (Fig. 4b, Additional file 5).

### Enzyme sensitivity of the immunomodulatory substances in LCM of *L. salivarius* strains B37 and B60 indicates differences in chemical nature

**Fig. 5:**
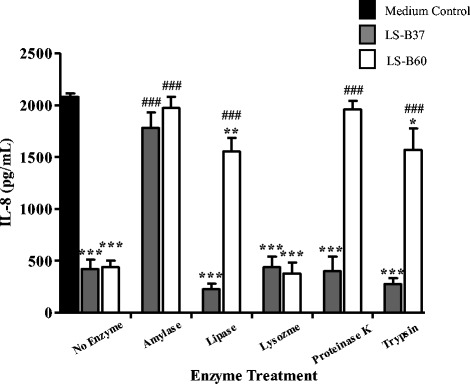
Enzyme sensitivity of the immunomodulatory substances in LCM of *L. salivarius.* Substances in LCM of LS-B37 and LS-B60 were incubated with enzymes α-amylase, lipase, lysozyme, proteinase K and trypsin and the effect on IL-8 production was tested using a Quantikine Human IL-8 Immunoassay. The experiments were performed three times in triplicate and the results represented the mean ± SEM, *p < 0.05, **p < 0.01, ***p < 0.001 as compared to medium control, ^###^ *p* < 0.001 as compared to non-treated LCM

To determine the chemical nature of IL-8 inhibitory substances in LS-B37 and LS-B60 LCM, treatments with α-amylase, lipase, proteinase K, trypsin, and lysozyme were performed. LCM from LS-B37 and LS-B60 were incubated with each of these enzymes for 6 h and then tested for *H. pylori*-induced IL-8 production. Treatment of LS-B37 LCM with α-amylase abolished IL-8 suppression as compared to media control ( *p* > 0.066) and non-treated LCM (>4 fold change, *p* < 0.0001), while treatment with lipase, proteinase K, trypsin, and lysozyme did not affect IL-8 suppression (Fig. 5, Additional file 6). Treatment of LS-B60 LCM with either α-amylase or proteinase K resulted in the loss of IL-8 inhibitory activity as compared to media control (*p* > 0.1003 and *p* > 0.0972, respectively) and non-treated LCM (>4 fold change, *p* < 0.001 and *p* < 0.001, respectively). The treatment with lipase or trypsin affected IL-8 suppression of LS-B60 LCM by 4-fold as compared to non-treated LCM (*p* < 0.001 and *p* < 0.001, respectively) although LS-B60 LCM retained some suppression of IL-8 production that was statistically different from media control (*p* < 0.01 and *p* < 0.05, respectively). No effect on IL-8 suppression was detected when LS-B60 LCM was treated with lysozyme (Fig. 5, Additional file 6). These results suggest that the inhibitory substance of LS-B37 is polysaccharide, whereas the one of LS-B60 is complex consisting of components of polysaccharide, protein and lipid. On the other hand, LS-B60 may produce multiple immunomodulating substances like glycoproteins or lipoproteins. As lysozyme had no effect on IL-8 suppression by LCM from either strain, the peptidoglycan of these lactobacilli is most likely not involved.

## Discussion

Persistent colonization of *H. pylori* results in chronic inflammation which predisposes infected people to severe peptic ulcer disease and gastric cancer [30]. Among several factors, pro-inflammatory cytokines and chemokines are the main mediators of mucosal inflammation and damage [4, 5]. IL-8 is a key chemokine in neutrophil infiltration [7, 8] that is associated with disease severity [14, 16] and considered a potential prognostic and predictive cancer biomarker [31]. In fact, whole genome analysis of *H. pylori*-exposed AGS gastric epithelial cells revealed that after a 3 h exposure IL-8 is the single most up-regulated gene among > 38,000 genes tested [32].

Specific strains of *Lactobacillus* have been shown to diminish *H. pylori*-induced IL-8 production in vitro and in vivo [23–25]. We have identified anti-inflammatory *Lactobacillus* spp. which suppress IL-8 secretion by *H. pylori*-induced AGS cells. Among these strains, it is interesting that *L. salivarius* strains B37 and B60 did not suppress IL-8 gene transcription at 4 h of co-culture [26]. It was previously reported that *L. casei* VSL#3 suppressed TNF-induced secretion of the T-cell chemokine interferon-inducible protein (IP-10) secretion by Mode-K intestinal epithelial cells without inhibiting IP-10 gene transcription. Study of the mechanism underlying this observation revealed that *L. casei* impairs vesicular pathways important for the secretion of IP-10 and the chemokine is subsequently degraded [33]. In an attempt to characterize the mechanism involved in the suppression of IL-8 secretion by LS-B37 and LS-B60, we first investigated the effect on IL-8 gene expression by LCM of LS-B37 and LS-B60 at various time points. Our data demonstrated that both LCM reduced IL-8 gene expression as early as a 2 h co-culture and the reduction by LS-B37 LCM continued at 4 h. Interference with IL-8 gene expression by probiotic microbes has been reported to occur at different time points of co-culture. *L. gasseri* OLL2716 (LG21) inhibited *H. pylori*-induced IL-8 gene expression by MKN45 gastric epithelial cells only at 6 h of co-culture [25]. On the contrary, *Saccharomyces boulardii* reduced IL-1β-induced IL-8 mRNA levels in HT-29 colonic epithelial cells at all time points studied from 0.5 to 4 h [34]. This study also demonstrated that LCM from LS-B37 and LS-B60 suppressed the activation of NF-κB at different time points (15 min and 2 h, respectively) but did not interfere with p-c-Jun activation. It has been demonstrated that IL-8 gene transcription in gastric epithelial cells requires the activation of both NF-κB and AP-1 [35], and a mutation in the IL-8 gene at the NF-κB binding site alone results in the inhibition of IL-8 gene expression [35, 36]. Therefore, suppression of only NF-κB activation by LCM of LS-B37 and LS-B60 should be sufficient for inhibiting IL-8 production by these lactobacilli. Anti-inflammatory *L. salivarius* B101 and *L. rhamnosus* B103 were also shown to suppress only p-NF-κB activated by *H. pylori* but the suppression occurred at various time points [26].

With an increase in the interest of anti-inflammatory *Lactobacillus* for *H. pylori* treatment, the molecular nature of immunomodulating substances has been investigated in *Lactobacillus* spp. Among several factors influencing host immune response, exopolysaccharides, which are extracellular polysaccharides attached to the bacterial cell surface or secreted into the extracellular environment, have been recognized to influence immune signaling [37]. In this study, the IL-8-suppressing substance in LCM of LS-B37 was a heat-stable, α-amylase -sensitive factor with a molecular size >100 kDa suggesting the production of an immunomodulatory exopolysaccharide by LS-B37. Exopolysaccharides of beneficial bacteria have long been shown to act as immunomodulating agents and their contributions to immune stimulation and immune suppression have been investigated in various aspects. Soluble polysaccharides with molecular weights between 60 and 2460 kDa of *Bifidobacterium adolescentis* M101-4 were found to stimulate the proliferation of murine splenocytes, suggesting their immunomodulation activity [38]. Polysaccharide-peptidoglycan complex derived from *Lactobacillus casei* strain Shirota inhibited the release of IL-6 in LPS-stimulated large intestinal lamina propria mononuclear cells isolated from mice with chronic colitis and in RAW264.7 cells in vitro [39]. Pretreatment of exopolysaccharides obtained from *Bifidobacterium longum* BCRC 14634 suppressed LPS-induced cell growth inhibition and release of TNF-α from J774A.1 macrophages [40]. Exopolysaccharides with molecular weights ≤ 70 kDa obtained from the culture medium of *Lactobacillus confusus* TISTR 1498 were observed to induce the production of nitric oxide and cytokines TNF-α, IL-1β, IL-6 and IL-10 through the activation of NF-κB and JNK pathways [41]. In addition, an exopolysaccaride of *Lactobacillus rhamnosus* GG alleviated inflammatory cytokines IL-6, IL-12 and TNF-α in LPS-induced IPEC-J2 porcine intestinal epithelial cells at mRNA level by attenuation of MAPK and NF-kB signaling activations [42].

On the contrary to LS-B37, LS-B60 LCM is sensitive to α-amylase, lipase, proteinase K and trypsin albeit with different magnitudes. It is probable that immunomodulatory substance(s) in LCM of LS-B60 is complex consisting of components of polysaccharide, protein and lipid. However, our result could not exclude the possibility that LS-B60 produces multiple immunomodulatory substances such as glycoproteins and lipoproteins. Bacterial glycoproteins have been identified [43] and secreted glycoproteins were reported in *L. plantarum* WCFS1 [44] and *L. rhamnosus* GG [45]. Glycoproteins of *L. plantarum* WCFS1 are thought to be recognized by the DC-specific ICAM-3-grabbing non-integrin (DC-SIGN) receptor and thereby eliciting specific cytokine production and immune responses in immature human dendritic cells [44]. Anti-inflammatory activity of cell-associated glycoprotein was recently reported in *L. plantarum* L67 for the protection of inflammation caused by cadmium ion in RAW 264.7 cells by suppressing the expression of AP-1 (c-Jun and c-Fos), mitogen-activated protein kinases (ERK, JNK and p38), and inducible nitric oxide synthase [46]. The immunomodulatory effect of bacterial lipoprotein (BLP) was also demonstrated in *E. coli* Nissle 1917 in that BLP acts like the conditioned medium in the modulation of T-cell cycle progression by binding to toll-like receptor-2 which may downregulate the expansion of newly recruited T cells into the mucosa and limit intestinal inflammation [47].

The structure and composition of polysaccharides and other biological substances are diverse among microbes and this variation may contribute to the different immunomodulation of host immune responses. Although we have not performed purification of bioactive substances, our initial characterization suggests the nature of different anti-inflammatory substances produced by LS-B37and LS-B60. Further studies are required to determine the exact molecular nature and function of these substances in the modulation of immune response to *H. pylori* infection.

## Conclusions

In this study, we have provided evidence that human gastric-derived *L. salivarius* strains B37 and B60 produce anti-inflammatory substances that suppress IL-8 production by *H. pylori*-induced AGS cells through the attenuation of NF-κB activation. Large, heat-stable bioactive substances produced by these two strains differ in enzymatic sensitivity. The immunomodulatory substance of *L. salivarius* B37 is similar to a polysaccharide, while that of LS-B60 is more structurally complex or includes multiple components such as glycoprotein and lipoprotein. These lactobacilli have potential as probiotics and their immunomodulatory substances may be pharmacologic agents for treatment of *H. pylori*-associated diseases.

## Methods

### Bacterial strains, cell line and culture conditions

*Lactobacillus salivarius* strains B37, B60, and B78 previously isolated from gastric biopsies of dyspeptic patients [48] were obtained from the stock culture maintained at the Department of Microbiology, Faculty of Medicine, Chulalongkorn University. Bacterial stock cultures were stored at −80 °C in deMan Rogosa Sharpe (MRS) broth (Oxoid, Hampshire, UK) containing 10 % (vol/vol) glycerol. All lactobacilli were cultured on MRS agar in an anaerobic chamber (Concept Plus, Ruskinn Technology, UK) (10 % CO_2_, 10 % H_2_, and 80 % N_2_) at 37 °C for 24 h. *Helicobacter pylori* ATCC 43504 (ATCC, Manassas, VA, USA) was grown on Columbia agar (Oxoid, England) supplemented with 7 % (v/v) horse serum (Gibco New Zealand Ltd, Auckland, New Zealand) and 7 % (v/v) sheep blood at 37 °C for 48 h under microaerophilic conditions ( 6–12 % O_2_, 5–8 % CO_2_) using gas generation sachets (Anaero Pack-MicroAero, Mitsubishi Gas Chemical, Japan) AGS human gastric adenocarcinoma epithelial cells (ATCC CRL-1739) obtained from the American Type Culture Collection (Manassas, VA, USA) were cultured as a monolayer (>80 % confluence) in RPMI 1640 medium supplemented with 10 % (v/v) heat-inactivated fetal bovine serum (Gibco-Invitrogen, USA) at 37 °C and 5 % CO_2_ atmosphere for 48 h. AGS cells were passaged every 2–3 days. Adherent cells were detached from the flask with 0.25 % (v/v) trypsin in 1 mM EDTA (Gibco-Invitrogen, USA), resuspended in fresh medium and used in the co-culture assay.

### Preparation of *Lactobacillus* conditioned media

*Lactobacillus-*conditioned media (LCM) were prepared as previously described [49]. *L. salivarius* strains were first cultured for 24 h, then adjusted to an OD600 of 0.1 in MRS broth and incubated anaerobically for 48 h. Cell free supernatants were collected by centrifugation at 4000 × g for 10 min at 4 °C, filtered through a 0.22 μm membrane filter (Minisart, Sartorius Stedim Biotech GmbH, Goettingen, Germany) and concentrated by speed vacuum drying for 3 h. (Savant instruments, Farmingdale, NY). Cell-free concentrated pellets were resuspended in an equal volume of RPMI 1640 medium (Gibco-Invitrogen, Carlsbad, CA, USA) and stored at −20 °C until further use.

### Effect of LCM treatment on IL-8 production in the co-culture assay

AGS gastric epithelial cells were treated with LCM and co-cultured with *H. pylori* to induce IL-8 production as previously described [50]. AGS cells (2.0 × 10^4^ cells/well) were pre-incubated as described above for 24 h. The culture supernatant was replaced with fresh RPMI medium containing either 5 % (v/v) LCM alone or in combination with viable *H. pylori* ATCC 43504 (6.0 × 10^6^ CFU/well) and incubated under 5 % CO_2_ at 37 °C for 24 h. Cell culture supernatants were collected by centrifugation at 125 × g for 7 min at 4 °C. IL-8 concentrations in culture supernatants were determined using the Quantikine Human IL-8 Immunoassay Kit (R&D Systems, Minneapolis, MN) according to the manufacturer’s instructions. All experiments were tested at least three times in triplicate.

### Analysis of IL-8 gene expression by qRT-PCR

The effects of LCM on the transcription of IL-8 in *H. pylori*-treated AGS epithelial cells were determined by qRT- PCR as previously described [26, 51] with minor modifications. AGS cells (5 × 10^5^cells/well) were cultured in a 24-well plate as described above and treated with viable *H. pylori* ATCC 43503 (1.5 × 10^8^ CFU/well) with or without LCM (5 % v/v) for 2, 4, and 6 h. Cell culture supernatants were removed by centrifugation and total RNA of treated AGS cells was extracted with TRIzol reagent (Invitrogen, USA) according to the manufacturer’s instructions. Complementary DNA (cDNA) was reverse transcribed from 10 ng total RNA using the SuperScript® VILO^TM^ cDNA Synthesis kit (Invitrogen, USA), and qPCR was performed in a LightCycler® 2.0 (Roche, Germany) for 45 cycles of: 10 s at 95 °C, 10 s at 65 °C, and 25 s at 72 °C. The following primers were used to amplify cDNA fragments: IL-8 forward primer (5′-ACACTGCGCCAACACAGAAATTA-3′), and IL-8 reverse primer (5′-TTTGCTTGAAGTTTCACTGGCATC-3′); Human glyceraldehyde-3-phosphate dehydrogenase (GAPDH) forward primer (5′-GCACCGTCAAGGCTGAGAAC-3′), and GAPDH reverse primer (5′-ATGGTGGTGAAGACGCCAGT-3′). IL-8 gene expression, relative to GAPDH, was calculated according to the 2^-ΔΔCp^ method [52].

### Examination of cell signaling pathways by quantitative western blot

The modulation of signaling pathways by LCM was examined by western blot as previously described with minor modifications [26, 28]. AGS cells (2.0 × 10^6^cells/well) were cultured in a 6-well plate as described above. AGS cells were stimulated with *H. pylori* ATCC 43504 (6.0 × 10^8^ CFU/well) in the presence or absence of LCM (5 % v/v) for 0.25, 0.5, 1, 2, and 3 h. Proteins were extracted from whole cell lysates of AGS-treated cells using Mammalian Protein Extraction Reagent (M-PER, Pierce Biotechnology, Illinois, USA) supplemented with Halt protease and phosphatase inhibitors (Pierce Biotechnology, Illinois, USA) according to the manufacturer’s instructions. Protein concentrations from cell lysates were determined using the Pierce® BCA protein assay kit (Pierce Biotechnology, Illinois, USA). Cell extracts were fractionated by 10 % sodium dodecyl sulfate polyacrylamide gel electrophoresis (SDS-PAGE), transferred onto PVDF membranes (Bio-Rad, Philadelphia, USA) and blocked with 10 % non-fat milk in TBST (50 mM Tris, pH 7.5, 150 mM NaCl, 0.05 % Tween 20). Blocked membranes were incubated with mouse antibodies against NF-κB (p65), phospho-NF-κB (p65), c-Jun, phospho-c-Jun (Santa Cruz Biotechnology, California, USA) and β-actin (Cell signaling Technology, Inc, MA, USA), washed with TBST and incubated with horseradish peroxidase-labeled goat anti-mouse secondary antibodies for 1 h. Peroxidase signals were measured and imaged by ChemiDoc™ XRS (Bio-Rad, Philadelphia, USA). Densitometric analyses for protein quantification were carried out using ImageJ 1.45 s software.

### Characterization of IL-8 suppressing substance(s) in LCM

The properties of IL-8-suppressing substances produced by *L. salivarius* strains were tested for heat stability, size estimation, and enzyme sensitivity. Heat stability was assessed by heating LCM to 100 °C for 0.25, 0.5, 1, and 2 h. The sizes of active factors present in LCM were estimated by 3 kDa, 50 kDa and 100 kDa Amicon® Ultra-4 Centrifugal Filters (Millipore Ireland B.V.*,*Tullagreen*,* Carrigtwohill*,* County Cork*,* Ireland) according to the manufacturer’s instruction. Enzyme sensitivity of LCM was tested by incubation with various enzymes: α-amylase in 20 mM sodium acetate, 7 mM sodium chloride (pH 6.9); lipase and lysozyme each in 50 mM Tris–HCl (pH 7.2); proteinase K and trypsin each in 50 mM Tris–HCl (pH 7.5–7.6). Each enzyme (Sigma, USA) was used at a final concentration of 1 mg/mL and incubated at 37 °C for 6 h, except for amylase and lysozyme which were incubated at 25 °C. Enzymes were inactivated by heating each enzyme-treated LCM at100 °C for 10 min. All treated samples were tested for IL-8 suppressive activity in the co-culture assay described above.

### Statistical analysis

All experiments were performed three times each in triplicate and the results were reported as mean ± standard deviation (SD) or standard error of mean (SEM). The data were analyzed in GraphPad Prism 5 using the unpaired t test with one-tailed distribution and considered statistically significant at a *p*-value ≤ 0.05, unless otherwise stated.

## Additional files

Additional file 1: Raw data used to generate Fig. 1. (DOCX 16 kb)
Additional file 2: Raw data used to generate Fig. 2. (DOCX 14 kb)
Additional file 3: Figure S1. Representative result of Western blot analysis on suppressive effects of LCM of LS-B37 and LS-B60 on *H. pylori*-activated NF-κB in AGS cells; **Table S1** Relative levels of p- NF-κB and NF-κB at various time points. (DOCX 241 kb)
Additional file 4: Table S2A. Raw data used to generate Fig. 4a showing the effect of heat treatment on LCM of LS-B37; **Table S2B** Raw data used to generate Fig. 4a showing the effect of heat treatment on LCM of LS-B60. (DOCX 20 kb)
Additional file 5: Table S3A. Raw data used to generate Fig. 4b showing the effect of size fractionation on LCM of LS-B37; **Table S3B** Raw data used to generate Fig. 4b showing the effect of size fractionation on LCM of LS-B60. (DOCX 21 kb)
Additional file 6: Table S4A. Raw data used to generate Fig. 5 showing the effect of enzyme treatment on LCM of LS-B37; **Table S4B** Raw data used to generate Fig. 5 showing the effect of enzyme treatment on LCM of LS-B60. (DOCX 21 kb)

## Abbreviations

ELISA: Enzyme-linked immunosorbent assay
IL-8: Interleukin-8
LCM: *Lactobacillus* conditioned medium
LS: *Lactobacillus salivarius*
MRS: deMan Rogosa Sharpe
NF-κB: Nuclear factor-kappa B
qRT-PCR: Quantitative reverse transcription polymerase chain reaction
SDS-PAGE: Sodium dodecyl sulfate polyacrylamide gel electrophoresis
