# Supplementary material for: Characterization of Lactobacillus salivarius strains B37 and B60 capable of inhibiting IL-8 production in Helicobacter pylori-stimulated gastric epithelial cells
Source: BMC Microbiol. 2016 Oct 18;16:242. doi: 10.1186/s12866-016-0861-x (PMC5070129; doi:10.1186/s12866-016-0861-x)
Supplement: Additional file 1: — Raw data used to generate Fig. 1. (DOCX 16 kb) [file 12866_2016_861_MOESM1_ESM.docx]

**Additional file 1: Raw data used to generate Fig. 1**

| Experiment | IL-8 concentration (pg/mL) | | | | | | | |
| --- | --- | --- | --- | --- | --- | --- | --- | --- |
|  | No *H. pylori* | | | | Plus *H. pylori* | | | |
|  | Medium Control | LS-B37 | LS-B60 | LS-B78 | Medium Control | LS-B37 | LS-B60 | LS-B78 |
| 1 | 77.35 | 99.73 | 110.88 | 81.90 | 1720.20 | 788.68 | 785.25 | 1857.39 |
|  | 123.49 | 78.04 | 144.35 | 89.33 | 2159.63 | 1161.91 | 946.45 | 2014.65 |
|  | 84.80 | 94.10 | 115.33 | 85.60 | 2129.54 | 1352.33 | 1076.53 | 1648.05 |
| 2 | 80.69 | 118.74 | 66.16 | 129.31 | 2086.00 | 1544.54 | 880.82 | 2042.19 |
|  | 82.56 | 129.03 | 96.45 | 153.70 | 1907.90 | 1717.64 | 1283.19 | 1992.44 |
|  | 123.49 | 129.03 | 96.83 | 145.45 | 2159.63 | 838.29 | 718.59 | 2016.68 |
| 3 | 77.93 | 85.68 | 93.70 | 129.31 | 2173.89 | 1741.53 | 1780.42 | 1857.39 |
|  | 111.16 | 99.51 | 110.44 | 129.31 | 1948.79 | 156.01 | 1656.54 | 2014.65 |
|  | 111.16 | 99.97 | 129.64 | 145.45 | 1206.24 | 1413.71 | 1641.52 | 1648.05 |
| Average | 96.96 | 103.76 | 107.09 | 121.04 | 1943.54 | 1190.52 | 1196.59 | 1899.05 |
| SD | 19.93 | 18.14 | 22.54 | 27.97 | 315.46 | 517.13 | 408.08 | 157.82 |
