# Supplementary material for: Characterization of Lactobacillus salivarius strains B37 and B60 capable of inhibiting IL-8 production in Helicobacter pylori-stimulated gastric epithelial cells
Source: BMC Microbiol. 2016 Oct 18;16:242. doi: 10.1186/s12866-016-0861-x (PMC5070129; doi:10.1186/s12866-016-0861-x)
Supplement: Additional file 2: — Raw data used to generate Fig. 2. (DOCX 14 kb) [file 12866_2016_861_MOESM2_ESM.docx]

**Additional file 2: Raw data used to generate Fig. 2**

| Experiment | Fold change in IL-8 gene expression (LCM/medium control) | | | | | | | | |
| --- | --- | --- | --- | --- | --- | --- | --- | --- | --- |
|  | 2 h | | | 4 h | | | 6h | | |
|  | Medium Control | LS-B37 | LS-B60 | Medium Control | LS-B37 | LS-B60 | Medium Control | LS-B37 | LS-B60 |
| 1 | 1.06 | 0.69 | 0.52 | 0.8 | 0.51 | 0.93 | 1.24 | 0.69 | 0.75 |
|  | 1.01 | 0.42 | 0.56 | 1.13 | 0.57 | 0.76 | 0.84 | 1.07 | 0.95 |
|  | 0.94 | 0.35 | 0.5 | 1.11 | 0.39 | 0.98 | 0.96 | 1.09 | 1.13 |
| 2 | 1.09 | 0.72 | 0.84 | 1.14 | 1.08 | 0.95 | 0.63 | 0.89 | 0.89 |
|  | 0.94 | 0.49 | 0.78 | 0.96 | 0.78 | 0.87 | 1.34 | 0.6 | 0.88 |
|  | 0.97 | 0.78 | 0.68 | 0.92 | 0.76 | 1.07 | 1.19 | 0.6 | 0.97 |
| 3 | 1.00 | 0.88 | 0.91 | 0.69 | 0.75 | 1.03 | 0.68 | 1.13 | 0.83 |
|  | 0.95 | 0.5 | 0.71 | 1.13 | 0.55 | 0.71 | 1.13 | 0.65 | 1.11 |
|  | 1.05 | 0.47 | 0.59 | 1.28 | 0.51 | 0.55 | 1.3 | 0.82 | 1.25 |
| Average | 1.00 | 0.59 | 0.68 | 1.02 | 0.66 | 0.87 | 1.03 | 0.84 | 0.97 |
| SD | 0.06 | 0.18 | 0.15 | 0.19 | 0.21 | 0.17 | 0.27 | 0.22 | 0.16 |
