# Supplementary material for: Characterization of Lactobacillus salivarius strains B37 and B60 capable of inhibiting IL-8 production in Helicobacter pylori-stimulated gastric epithelial cells
Source: BMC Microbiol. 2016 Oct 18;16:242. doi: 10.1186/s12866-016-0861-x (PMC5070129; doi:10.1186/s12866-016-0861-x)
Supplement: Additional file 3: Figure S1. — Representative result of Western blot analysis on suppressive effects of LCM of LS-B37 and LS-B60 on H. pylori-activated NF-κB in AGS cells; Table S1 Relative levels of p- NF-κB and NF-κB at various time points. (DOCX 241 kb) [file 12866_2016_861_MOESM3_ESM.docx]

**Additional file 3:**

**Figure S1. Representative result of Western blot analysis on suppressive effects of LCM of LS-B37 and LS-B60 on *H. pylori*-activated NF-κB in AGS cells**


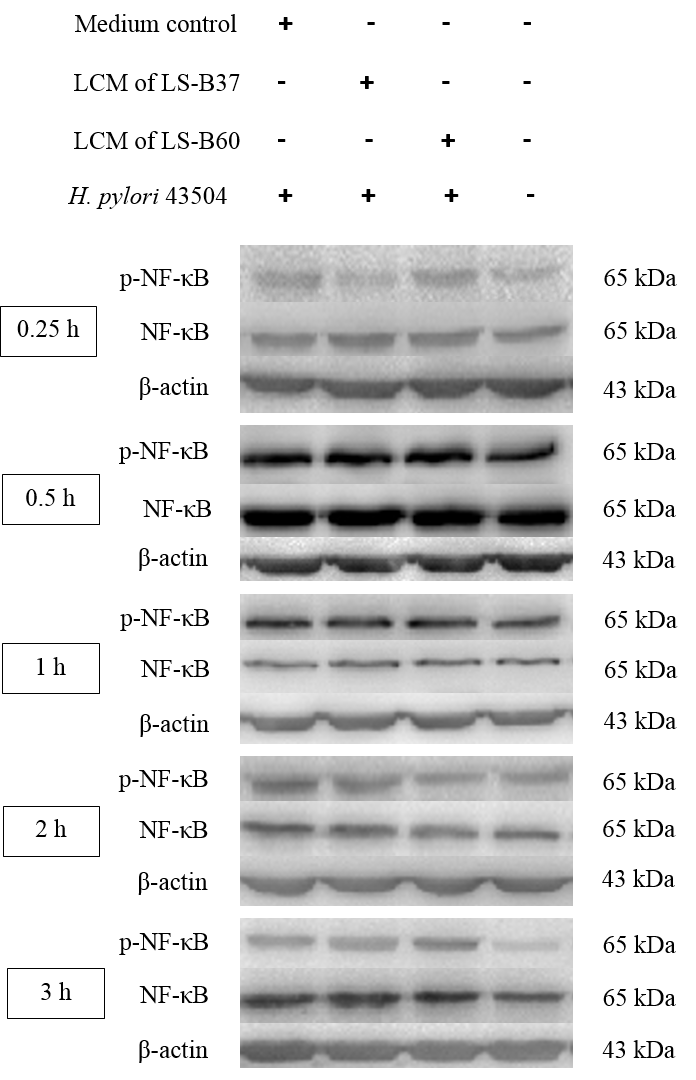


**Table S1. Relative levels of p-NF-κB and NF-κB at various time points**

| Time of incubation | Sample plus  *H. pylori* | p- NF-κB/NF-κB | | | Average | SD |
| --- | --- | --- | --- | --- | --- | --- |
|  |  | Exp. 1 | Exp. 2 | Exp. 3 |  |  |
| 0.25 h | Medium control | 0.96 | 0.98 | 1.16 | 1.03 | 0.11 |
|  | LS-B37 | 0.68 | 0.75 | 0.78 | 0.74 | 0.05 |
|  | LS-B60 | 0.82 | 1.04 | 0.93 | 0.93 | 0.11 |
| 0.5 h | Medium control | 0.968 | 0.920 | 1.109 | 0.999 | 0.098 |
|  | LS-B37 | 1.082 | 0.993 | 1.098 | 1.058 | 0.057 |
|  | LS-B60 | 0.953 | 0.841 | 0.935 | 0.910 | 0.060 |
| 1 h | Medium control | 0.77 | 1.10 | 0.98 | 0.95 | 0.17 |
|  | LS-B37 | 0.89 | 1.26 | 0.64 | 0.93 | 0.31 |
|  | LS-B60 | 0.56 | 1.28 | 0.76 | 0.86 | 0.37 |
| 2 h | Medium control | 1.09 | 1.17 | 1.21 | 1.16 | 0.06 |
|  | LS-B37 | 1.11 | 0.77 | 1.04 | 0.97 | 0.18 |
|  | LS-B60 | 0.59 | 0.66 | 0.90 | 0.71 | 0.16 |
| 3 h | Medium control | 0.85 | 1.02 | 1.06 | 0.98 | 0.11 |
|  | LS-B37 | 0.90 | 0.82 | 1.16 | 0.96 | 0.18 |
|  | LS-B60 | 0.52 | 1.04 | 1.03 | 0.86 | 0.30 |
