# Supplementary material for: Characterization of Lactobacillus salivarius strains B37 and B60 capable of inhibiting IL-8 production in Helicobacter pylori-stimulated gastric epithelial cells
Source: BMC Microbiol. 2016 Oct 18;16:242. doi: 10.1186/s12866-016-0861-x (PMC5070129; doi:10.1186/s12866-016-0861-x)
Supplement: Additional file 4: Table S2A. — Raw data used to generate Fig. 4a showing the effect of heat treatment on LCM of LS-B37; Table S2B Raw data used to generate Fig. 4a showing the effect of heat treatment on LCM of LS-B60. (DOCX 20 kb) [file 12866_2016_861_MOESM4_ESM.docx]

**Additional file 4:**

**Table S2A. Raw data used to generate Fig. 4A showing the effect of heat treatment on LCM of LS-B37**

| Experiment | IL-8 concentration (pg/mL) | | | | | |
| --- | --- | --- | --- | --- | --- | --- |
|  | Medium control | No heat | Heat treatment (h) | | | |
|  |  |  | 0.25 | 0.5 | 1 | 2 |
| 1 | 2286.43 | 408.02 | 1441.6 | 617.39 | 479.96 | 1307.23 |
|  | 2251.40 | 649.20 | 1000.01 | 343.86 | 359.86 | 981.55 |
|  | 2128.83 | 718.02 | 1050.38 | 815.58 | 304.33 | 977.91 |
| 2 | 1232.76 | 318.02 | 430.57 | 959.31 | 1147.21 | 187.90 |
|  | 1741.93 | 758.37 | 194.27 | 744.05 | 823.45 | 122.06 |
|  | 1504.70 | 284.64 | 878.45 | 716.95 | 199.95 | 357.00 |
| 3 | 1255.610 | 495.956 | 694.53 | 482.710 | 334.574 | 622.692 |
|  | 1174.520 | 368.498 | 876.23 | 429.092 | 828.453 | 365.482 |
|  | 1116.069 | 580.393 | 473.28 | 929.559 | 720.103 | 368.120 |
| Average | 1632.47211 | 509.013 | 782.147 | 670.945 | 577.543 | 587.772 |
| SD | 482.64864 | 175.907 | 378.269 | 218.573 | 316.511 | 411.371 |

**Table S2B. Raw data used to generate Fig. 4A showing** **the effect of heat treatment on LCM of LS-B60**

| Experiment | IL-8 concentration (pg/mL) | | | | | |
| --- | --- | --- | --- | --- | --- | --- |
|  | Medium control | No heat | Heat treatment (h) | | | |
|  |  |  | 0.25 | 0.5 | 1 | 2 |
| 1 | 1506.68 | 779.14 | 500.00 | 1289.42 | 124.46 | 201.79 |
|  | 1506.68 | 1084.64 | 519.97 | 1058.68 | 199.22 | 755.37 |
|  | 1611.79 | 468.06 | 405.90 | 1187.44 | 487.47 | 238.06 |
| 2 | 1442.36 | 917.18 | 645.65 | 352.71 | 1025.40 | 553.44 |
|  | 2194.53 | 632.09 | 385.19 | 426.48 | 943.73 | 384.81 |
|  | 2092.86 | 859.72 | 419.56 | 602.63 | 1025.40 | 243.65 |
| 3 | 1936.00 | 721.00 | 679.71 | 270.00 | 364.00 | 805.00 |
|  | 1675.00 | 281.00 | 170.77 | 612.00 | 61.00 | 1174.30 |
|  | 1545.00 | 631.00 | 679.71 | 1057.00 | 451.00 | 750.00 |
| Average | 1723.43 | 708.20 | 489.61 | 761.82 | 520.19 | 567.38 |
| SD | 279.11 | 241.21 | 166.63 | 387.99 | 385.78 | 330.52 |
