# Supplementary material for: Characterization of Lactobacillus salivarius strains B37 and B60 capable of inhibiting IL-8 production in Helicobacter pylori-stimulated gastric epithelial cells
Source: BMC Microbiol. 2016 Oct 18;16:242. doi: 10.1186/s12866-016-0861-x (PMC5070129; doi:10.1186/s12866-016-0861-x)
Supplement: Additional file 5: Table S3A. — Raw data used to generate Fig. 4b showing the effect of size fractionation on LCM of LS-B37; Table S3B Raw data used to generate Fig. 4b showing the effect of size fractionation on LCM of LS-B60. (DOCX 21 kb) [file 12866_2016_861_MOESM5_ESM.docx]

**Additional file 5:**

**Table S3A. Raw data used to generate Fig. 4B showing the effect of size fractionation on LCM of LS-B37**

| Experiment | IL-8 concentration (pg/mL) | | | | | | | |
| --- | --- | --- | --- | --- | --- | --- | --- | --- |
|  | Medium control | No fractionation | Size- fractionated LCM | | | | | |
|  |  |  | <3  kDa | >3  kDa | <50  kDa | >50  kDa | <100 kDa | >100 kDa |
| 1 | 1615.46 | 405.20 | 1831.19 | 617.39 | 1750.56 | 1482.34 | 2128.64 | 2120.23 |
|  | 1933.57 | 147.52 | 1797.16 | 343.86 | 1776.30 | 481.01 | 1620.30 | 581.15 |
|  | 1752.77 | 163.93 | 1814.13 | 815.58 | 1669.53 | 464.53 | 1684.04 | 605.87 |
| 2 | 1933.57 | 115.45 | 1677.75 | 959.31 | 1278.95 | 631.10 | 1898.60 | 1137.90 |
|  | 1954.10 | 232.68 | 1660.47 | 744.05 | 1533.19 | 596.10 | 1637.57 | 942.90 |
|  | 1691.38 | 495.91 | 1532.05 | 716.95 | 1457.08 | 634.80 | 1398.23 | 466.82 |
| 3 | 1691.38 | 149.53 | 1831.19 | 482.71 | 1371.01 | 631.10 | 2049.00 | 653.41 |
|  | 1603.19 | 151.10 | 1797.16 | 429.09 | 1500.63 | 596.10 | 2058.34 | 483.20 |
|  | 1393.97 | 164.63 | 1814.13 | 929.56 | 1627.84 | 634.80 | 2117.40 | 483.23 |
| Average | 1729.93 | 225.11 | 1750.58 | 670.94 | 1551.68 | 683.54 | 1843.57 | 830.19 |
| SD | 186.65 | 133.44 | 104.04 | 218.57 | 169.05 | 306.72 | 265.44 | 535.03 |

**Table S3B. Raw data used to generate Fig. 4B showing the effect of size fractionation on LCM of LS-B60**

| Experiment | IL-8 concentration (pg/mL) | | | | | | | |
| --- | --- | --- | --- | --- | --- | --- | --- | --- |
|  | Medium control | No fractionation | Size- fractionated LCM | | | | | |
|  |  |  | <3  kDa | >3  kDa | <50  kDa | >50  kDa | <100 kDa | >100 kDa |
| 1 | 1615.46 | 332.36 | 1662.64 | 1289.42 | 1613.33 | 916.66 | 2115.38 | 2042.29 |
|  | 1933.57 | 748.96 | 1965.69 | 1058.68 | 1482.34 | 707.10 | 1482.34 | 448.60 |
|  | 1752.77 | 892.10 | 1970.75 | 1187.44 | 1916.66 | 470.00 | 1916.66 | 562.91 |
| 2 | 1933.57 | 624.00 | 941.05 | 352.71 | 1964.52 | 483.80 | 1964.52 | 495.91 |
|  | 1954.10 | 274.20 | 1950.00 | 426.48 | 2128.64 | 331.30 | 2128.64 | 1239.24 |
|  | 1691.38 | 431.90 | 2009.00 | 602.63 | 2115.38 | 232.50 | 2115.38 | 1210.31 |
| 3 | 1691.38 | 243.91 | 1662.64 | 270.00 | 1602.94 | 738.00 | 2120.23 | 653.41 |
|  | 1603.19 | 160.95 | 1965.69 | 612.00 | 1656.75 | 763.20 | 1953.85 | 480.20 |
|  | 1393.97 | 162.52 | 1970.75 | 1057.00 | 1810.86 | 714.20 | 1248.28 | 433.23 |
| Average | 1729.93 | 430.1 | 1788.69 | 761.818 | 1810.16 | 595.196 | 1893.92 | 840.678 |
| SD | 186.655 | 265.82 | 345.286 | 387.987 | 235.17 | 225.808 | 316.177 | 550.014 |
