# Supplementary material for: Characterization of Lactobacillus salivarius strains B37 and B60 capable of inhibiting IL-8 production in Helicobacter pylori-stimulated gastric epithelial cells
Source: BMC Microbiol. 2016 Oct 18;16:242. doi: 10.1186/s12866-016-0861-x (PMC5070129; doi:10.1186/s12866-016-0861-x)
Supplement: Additional file 6: Table S4A. — Raw data used to generate Fig. 5 showing the effect of enzyme treatment on LCM of LS-B37; Table S4B Raw data used to generate Fig. 5 showing the effect of enzyme treatment on LCM of LS-B60. (DOCX 21 kb) [file 12866_2016_861_MOESM6_ESM.docx]

**Additional file 6:**

**Table S4A. Raw data used to generate Fig. 5 showing the effect of enzyme treatment on LCM of LS-B37**

| Experiment |  |  | IL-8 concentration (pg/mL) | | | | |
| --- | --- | --- | --- | --- | --- | --- | --- |
|  |  |  | Enzyme- treated LCM | | | | |
|  | Medium control | No enzyme | α-Amylase | Lipase | Lysozyme | Proteinase K | Trypsin |
| 1 | 1930.58 | 726.49 | 1784.73 | 413.95 | 68.72 | 77.94 | 239.96 |
|  | 2049.69 | 614.82 | 1772.92 | 506.2 | 364.57 | 326.61 | 93.10 |
|  | 2112.87 | 491.07 | 1693.73 | 128.26 | 104.84 | 109.95 | 235.33 |
| 2 | 2184.13 | 119.69 | 2270.19 | 413.95 | 879.24 | 284.99 | 576.59 |
|  | 2179.12 | 193.12 | 2301.11 | 138.11 | 370.05 | 121.29 | 235.33 |
|  | 2225.91 | 109.29 | 1180.63 | 108.14 | 859.27 | 554.63 | 408.73 |
| 3 | 2030.8 | 822.98 | 996.44 | 122.59 | 138.43 | 670.73 | 446.68 |
|  | 2024.71 | 409.55 | 2041.00 | 99.19 | 582.75 | 456.41 | 134.70 |
|  | 1990.38 | 321.39 | 2003.95 | 118.01 | 574.86 | 1017.07 | 132.38 |
| Average | 2080.91 | 423.16 | 1782.74 | 227.60 | 438.08 | 402.18 | 278.09 |
| SD | 99.8655 | 261.55 | 448.72 | 165.36 | 308.01 | 415.54 | 164.21 |

**Table S4B. Raw data used to generate Fig. 5 showing the effect of enzyme treatment on LCM of LS-B60**

| Experiment | IL-8 concentration (pg/mL) | | | | | | |
| --- | --- | --- | --- | --- | --- | --- | --- |
|  |  |  | Enzyme- treated LCM | | | | |
|  | Medium control | No enzyme | α-Amylase | Lipase | Lysozyme | Proteinase K | Trypsin |
| 1 | 1930.58 | 359.40 | 1410.90 | 1386.49 | 73.04 | 1961.37 | 2326.31 |
|  | 2049.69 | 371.03 | 1595.10 | 1822.60 | 197.74 | 2198.10 | 505.41 |
|  | 2112.87 | 447.48 | 1903.81 | 1194.22 | 619.36 | 1961.37 | 857.41 |
| 2 | 2184.13 | 507.53 | 2194.16 | 966.52 | 251.51 | 1961.37 | 2151.78 |
|  | 2179.12 | 896.53 | 2190.81 | 1725.51 | 226.00 | 2198.10 | 1261.91 |
|  | 2225.91 | 223.79 | 2301.11 | 2101.23 | 806.35 | 1961.37 | 1574.06 |
| 3 | 2030.80 | 359.40 | 2301.11 | 1152.41 | 281.28 | 1335.54 | 1653.65 |
|  | 2024.71 | 371.03 | 1826.42 | 1887.10 | 881.05 | 2074.67 | 1546.84 |
|  | 1990.38 | 447.48 | 2044.73 | 1754.08 | 70.88 | 1966.99 | 2255.01 |
| Average | 2080.91 | 442.63 | 1974.24 | 1554.46 | 378.58 | 1957.65 | 1570.26 |
| SD | 99.87 | 187.84 | 316.59 | 389.86 | 308.84 | 254.09 | 624.83 |
